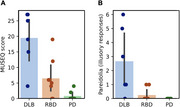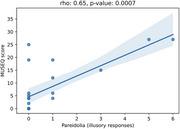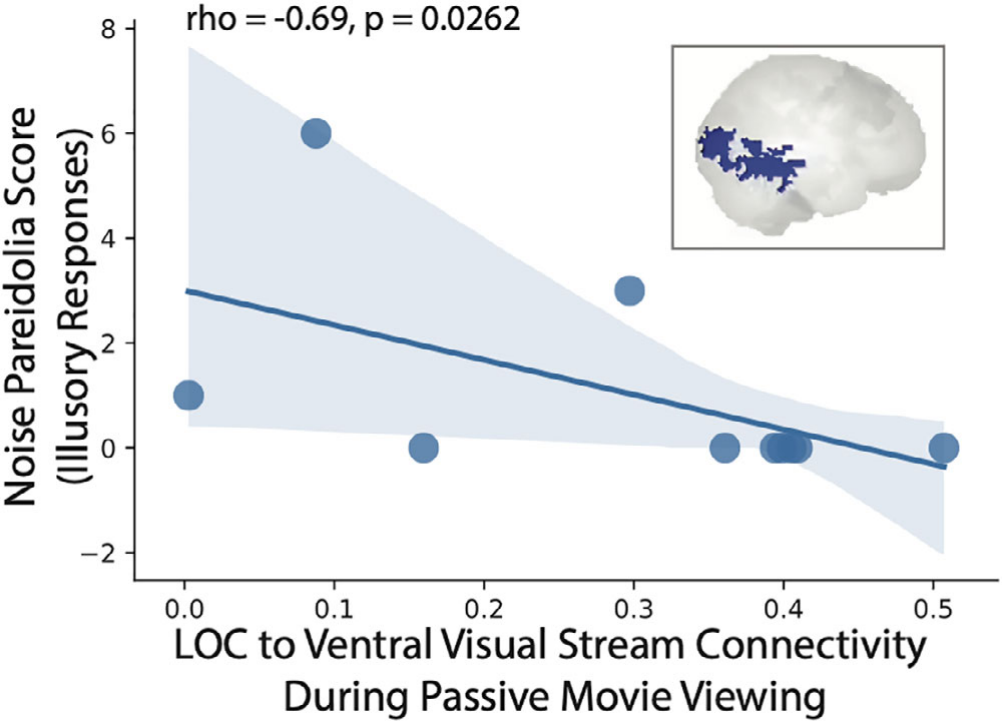# Neural mechanisms of visuoperceptual disturbances in Dementia with Lewy Bodies

**DOI:** 10.1002/alz70856_096478

**Published:** 2025-12-24

**Authors:** Raina Vin, Hae Young H Yi, Sophia Moret, Millie Lawrence, Veronica Santini, Jesse M Cedarbaum, Albert R Powers, Carolyn A Fredericks

**Affiliations:** ^1^ Yale University School of Medicine, New Haven, CT, USA; ^2^ Yale School of Medicine, New Haven, CT, USA

## Abstract

**Background:**

Dementia with Lewy Bodies (DLB) and Parkinson's Disease (PD) both arise from α‐synuclein aggregates and are often preceded by REM sleep behavior disorder (RBD). Unlike PD, DLB is marked by the early appearance of abnormal perceptual experiences such as presence and passage phenomena and illusions, followed by the onset of vivid visual hallucinations. Reliable markers predicting conversion from RBD to DLB/PD remain elusive. Unraveling the neural mechanisms underlying these perceptual abnormalities in DLB could pave the way for earlier diagnosis and targeted intervention.

**Method:**

Structural and functional MRI data was acquired from 18 participants (RBD: 8, DLB: 4, PD: 6) during a naturalistic movie‐viewing task. A spherical seed centered in the left lateral occipital cortex was defined, and seed‐based voxelwise connectivity across the whole brain was computed. Participants also completed the Noise Pareidolia Test, during which they viewed a series of black‐and‐white patterns, some with hidden human faces and others without. Illusory responses were quantified as the number of patterns in which participants incorrectly perceived a face. Additionally, the Multi‐Modality Unusual Sensory Experiences Questionnaire (MUSEQ) was used to characterize abnormal perceptual experiences and hallucinations across the three groups.

**Result:**

DLB participants exhibited higher mean MUSEQ scores (Cohen's *d* = 3.02) and more illusory responses on the Noise Pareidolia Test than PD (Cohen's *d* = 1.63). RBD participants displayed intermediate scores, with some showing scores near zero, resembling PD, and others demonstrating elevated scores similar to DLB. Across all groups, MUSEQ scores were significantly positively correlated with number of illusory responses (*r* = 0.65, *p* = 0.0007). Moreover, increased illusory responses were associated with reduced connectivity between the lateral occipital seed and a large ventral visual stream cluster during passive movie viewing (*r* = ‐0.69, *p* = 0.02).

**Conclusion:**

Abnormal perceptual experiences and performance on visuospatial tasks may help identify clinically relevant subgroups within prodromal RBD. Reduced bottom‐up‐driven visual connectivity associated with an increase in illusory percepts provides insight into the neural mechanisms of perceptual abnormalities in DLB and prodromal RBD. This offers potential for early detection and could inform the development of novel treatment strategies.